# Biological Evaluation of [^18^F]AlF-NOTA-NSC-GLU as a Positron Emission Tomography Tracer for Hepatocellular Carcinoma

**DOI:** 10.3389/fchem.2021.630452

**Published:** 2021-04-16

**Authors:** Liping Lin, Xianhong Xiang, Shu Su, Shaoyu Liu, Ying Xiong, Hui Ma, Gongjun Yuan, Dahong Nie, Ganghua Tang

**Affiliations:** ^1^Department of Radiology Intervention and Medical Imaging, Guangdong Engineering Research Center for Medical Radiopharmaceuticals Translational Application, The First Affiliated Hospital, Sun Yat-sen University, Guangzhou, China; ^2^Department of Radiotherapy, The First Affiliated Hospital, Sun Yat-sen University, Guangzhou, China; ^3^Nanfang PET Center, Department of Nuclear Medicine, Nanfang Hospital, Southern Medical University, Guangzhou, China

**Keywords:** hepatocellular carcinoma, PET, [^18^F]AlF-NOTA-NSC-GLU, system X_AG_^-^, tumor imaging

## Abstract

**Purpose:** N-(2-[^18^F]fluoropropionyl)-L-glutamate ([^18^F]FPGLU) for hepatocellular carcinoma (HCC) imaging has been performed in our previous studies, but its radiosynthesis method and stability *in vivo* need to be improved. Hence, we evaluated the synthesis and biological properties of a simple [^18^F]-labeled glutamate analog, [^18^F]AlF-1,4,7-triazacyclononane-1,4,7-triacetic-acid-2-S-(4-isothiocyanatobenzyl)-l-glutamate ([^18^F]AlF-NOTA-NSC-GLU), for HCC imaging.

**Procedures:** [^18^F]AlF-NOTA-NSC-GLU was synthesized via a one-step reaction sequence from NOTA-NSC-GLU. In order to investigate the imaging value of [^18^F]AlF-NOTA-NSC-GLU in HCC, we conducted positron emission tomography/computed tomography (PET/CT) imaging and competitive binding of [^18^F]AlF-NOTA-NSC-GLU in human Hep3B tumor-bearing mice. The transport mechanism of [^18^F]AlF-NOTA-NSC-GLU was determined by competitive inhibition and protein incorporation experiments *in vitro*.

**Results:** [^18^F]AlF-NOTA-NSC-GLU was prepared with an overall radiochemical yield of 29.3 ± 5.6% (*n* = 10) without decay correction within 20 min. *In vitro* competitive inhibition experiments demonstrated that the Na^+^-dependent systems ${\text{X}}_{\text{AG}}^{-}$, B0^+^, ASC, and minor ${\text{X}}_{\text{C}}^{-}$ were involved in the uptake of [^18^F]AlF-NOTA-NSC-GLU, with the Na^+^-dependent system ${\text{X}}_{\text{AG}}^{-}$ possibly playing a more dominant role. Protein incorporation studies of the Hep3B human hepatoma cell line showed almost no protein incorporation. Micro-PET/CT imaging with [^18^F]AlF-NOTA-NSC-GLU showed good tumor-to-background contrast in Hep3B human hepatoma-bearing mouse models. After [^18^F]AlF-NOTA-NSC-GLU injection, the tumor-to-liver uptake ratio of [^18^F]AlF-NOTA-NSC-GLU was 2.06 ± 0.17 at 30 min post-injection. *In vivo* competitive binding experiments showed that the tumor-to-liver uptake ratio decreased with the addition of inhibitors to block the X_AG_ system.

**Conclusions:** We have successfully synthesized [^18^F]AlF-NOTA-NSC-GLU as a novel PET tracer with good radiochemical yield and high radiochemical purity. Our findings indicate that [^18^F]AlF-NOTA-NSC-GLU may be a potential candidate for HCC imaging. Also, a further biological evaluation is underway.

## Introduction

Hepatocellular carcinoma (HCC) is among the leading causes of cancer-related deaths and is the fifth most frequently diagnosed malignancy worldwide (Wallace et al., 2015; Schutte et al., 2016). Also known as “the silent killer,” early-stage HCC is often missed, and the 5-year survival rate of an advanced HCC is <5%, compared to 40–70% if it is diagnosed early (El-Serag and Davila, 2011). Thus, timely diagnosis and precise staging are essential for selecting the correct treatment and improving prognosis. Increasingly, medical imaging has become the primary method for the non-invasive diagnosis of HCC, supported by guidelines from the American Association for the Study of Liver Diseases and the European Association for the Study of the Liver (Bruix and Sherman, 2011; European Association for Study of Liver, 2012). Notably, the most common examinations conducted for diagnosing HCC consist of computed tomography (CT) and magnetic resonance imaging (MRI) (Bruix and Sherman, 2011; European Association for the Study of the Liver, 2018). The sensitivities of these conventional imaging for HCC are high and still leave room for improvement, particularly for lesions smaller than 2 cm. Positron emission tomography/computed tomography (PET/CT) provides high image resolution and quality for HCC imaging, which is also a non-invasive imaging technique. It can detect and characterize tumors based on their molecular and biochemical properties (Momcilovic and Shackelford, 2018) and plays a vital role in the evaluation of HCC, especially with the rapid development of hepatocyte-specific PET tracers.

A non-invasive functional technique, 2-[^18^F]fluoro-2-deoxy-D-glucose ([^18^F]FDG) PET/CT has become the standard diagnostic procedure for various kinds of malignancies. Nevertheless, recent investigations, including clinical PET studies, have noted the significant false-positive rate of [^18^F]FDG PET, resulting from its failure to differentiate carcinogenesis from inflammation (Chang et al., 2006; Annunziata et al., 2014; Adams and Kwee, 2016). In addition, the sensitivity of [^18^F]FDG PET/CT for diagnosing HCC (50–55%) is less than satisfactory (Wolfort et al., 2010; Hayakawa et al., 2014). Hence, a more specific and sensitive PET imaging tracer is required.

Tumor cells can be identified by the abnormal proliferation and metabolic activities of nutrients, including glucose, amino acids (AAs), fatty acids, and vitamins (Plathow and Weber, 2008). To increase more specific tumor uptake, PET tracers for the metabolism of glucose, lipids, AAs, and nucleic acids in tumors have been developed. AA PET supplements glucose PET and plays a crucial role in oncologic imaging. Where glucose sources may be insufficient to sustain a rate of growth, some tumor cells are able to catabolize glutamine as a source of carbon via the glutaminolytic pathway. Furthermore, studies have shown that glutaminolysis may be another metabolic pathway in [^18^F]FDG-negative tumors (Rajagopalan and DeBerardinis, 2011). On the one hand, glutamate can be converted to glutamine. On the other hand, glutamate plays a fundamental role in the metabolism of AAs (Brosnan and Brosnan, 2013).

Studies have supported the use of ^18^F-labeled AAs in the imaging of various tumors (e.g., gliomas, neuroendocrine tumors, prostate cancer, and breast cancer) (McConathy et al., 2012; Ulaner et al., 2017). L-methyl-[^11^C]methionine ([^11^C]Met) is the most commonly used AA tracer in HCC imaging. However, the sensitivity and specificity of [^11^C]Met for HCC are inadequate (Kuang et al., 2014a,b). Moreover, the short half-life of carbon-11 also limits the adoption of [^11^C]Met PET (Park et al., 2008; Hwang et al., 2009; Cheung et al., 2013). In recent years, dual-tracer ([^18^F]FDG and [^11^C]acetate) protocols have been introduced for HCC imaging, with improved sensitivity and specificity (Hwang et al., 2009; Li et al., 2017). Unfortunately, dual-tracer PET/CT incurs more radiation burden than single-tracer PET/CT, which limits its clinical application (Ho et al., 2007; Liu et al., 2016).

Currently, we are working on a series of radiolabeled N-substituted AA analogs, which target the increased levels of AA transport by various types of malignant cells (e.g., systems L, ${\text{X}}_{\text{AG}}^{-}$, ${\text{X}}_{\text{C}}^{-}$, ASC, and A), as potential PET tracers for imaging HCC (Sun et al., 2017). Some reports have indicated that N-(2-[^18^F]fluoropropionyl)-L-glutamic acid ([^18^F]FPGLU) is a useful PET agent with relatively better detection of cancer, compared to [^18^F]FDG via a two-step reaction sequence (Hu et al., 2014; Sun et al., 2017). A simple one-step procedure, which prepares ^18^F-labeled peptides by chelating an aluminum fluoride (AlF) with 1,4,7-triazacyclononane-1,4,7-triacetic-acid (NOTA), provides a novel strategy to simplify the labeling procedure (McBride et al., 2009, 2010). This prompted the design of a simple ^18^F-labeled AA tracer.

In this study, we produced a small-molecule ^18^F-labeled AA tracer ([^18^F]AlF-NOTA-NSC-GLU) with direct labeling via AlF chelation. We also evaluated the value of [^18^F]AlF-NOTA-NSC-GLU with biodistribution, transport assays *in vitro*, PET imaging of HCC Hep3B-bearing mice, and competitive binding properties *in vivo*.

## Materials and Methods

### General Information

All chemicals used in the synthesis were commercially sourced and used without further purification unless otherwise indicated. [^18^F]FDG was radiolabeled as previously described (Luo et al., 2009). Sep-Pak light QMA and Plus C18 cartridges were purchased from Waters Corporation (Milford, MA, USA). Sep-Pak light QMA cartridges were preconditioned with 10 ml of NaHCO_3_ aqueous (8.4%) and water in advance. Preconditioning of the Plus C18 cartridge was performed with 10 ml of ethanol followed by 10 ml of water. A micro PET/CT scanner by Siemens Healthineers (Erlangen, Germany) was used.

### Animal Models and Cell Culture

The human HCC Hep3B cell line was provided by Stem Cell Bank, Chinese Academy of Sciences (Shanghai, China). The cells were cultivated in Dulbecco's Modified Eagle Medium (DMEM), containing 10% fetal bovine serum (Gibco, Grand Island, NY, USA) and 1% penicillin streptomycin at 37°C in a humidified atmosphere of 5% CO_2_ and 95% air.

Male BALB/c nude mice (4–6 weeks old and weighing 18–22 g) were purchased from Beijing Vital River Laboratory Animal Technology Co. Ltd. (Beijing, China). HCC Hep3B cells (1–2 × 10^7^) were subcutaneously implanted in the left or right axilla and allowed to grow for 2–3 weeks. BALB/c nude mice underwent imaging when their tumors had grown to 10–15 mm in diameter.

All the animal experiments were conducted in accordance with the recommendations and guidelines of the Institutional Animal Care and Utilization Committee (IACUU) of the First Affiliated Hospital, Sun Yat-Sen University (approval number 2018033). All animals were housed, five animals per cage, under standard laboratory conditions.

### Synthesis of [^18^F]AlF-NOTA-NSC-GLU

**Figure 1:**
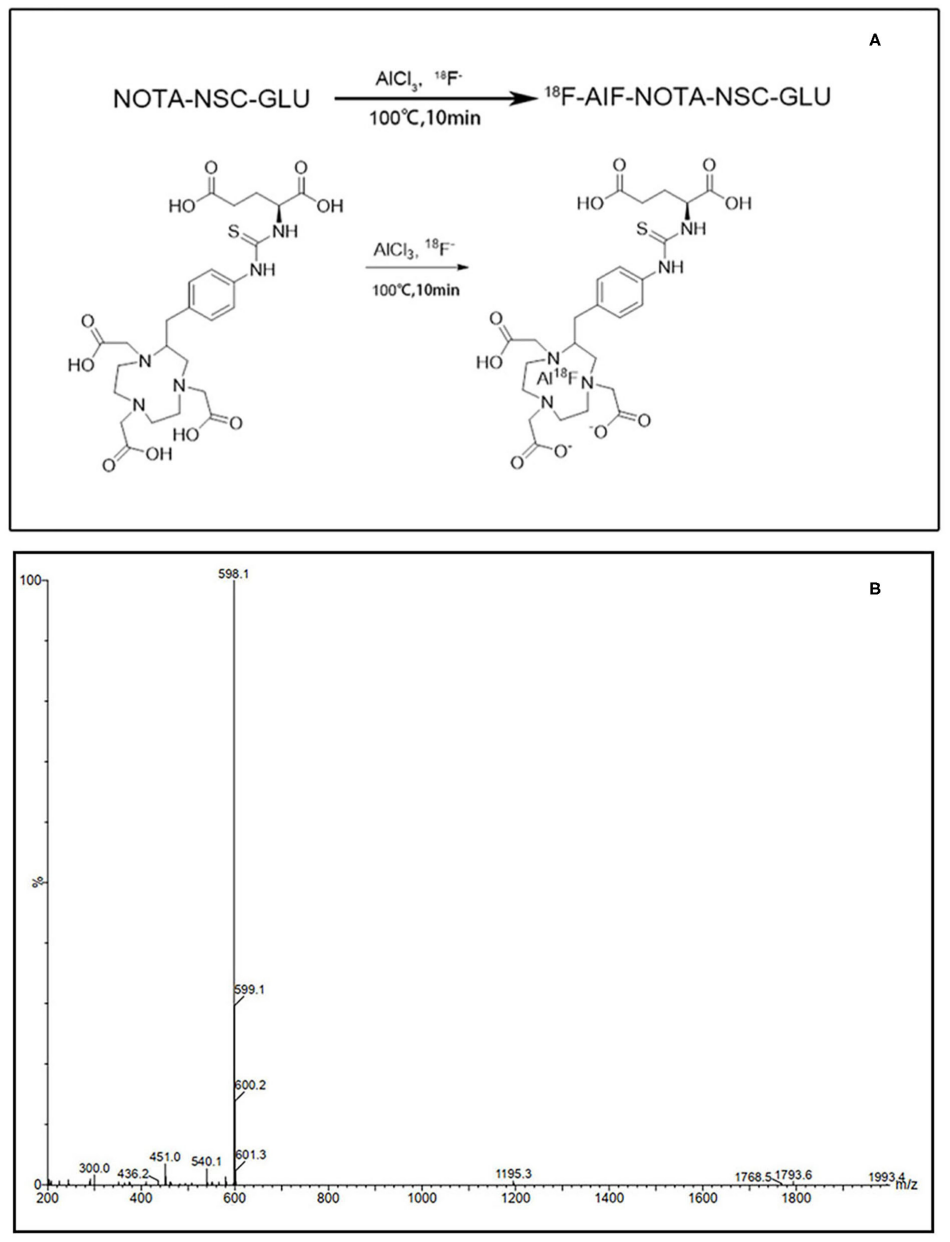
The synthetic scheme and MS spectra of [^18^F]AlF-NOTA-NSC-GLU. **(A)** The synthetic scheme of [^18^F]AlF-NOTA-NSC-GLU. **(B)** The MS spectra of [^18^F]AlF-NOTA-NSC-GLU. [^18^F]AlF-NOTA-NSC-GLU, [^18^F]-aluminum-fluoride-1,4,7-triazacyclononane-1,4,7-triacetic-acid-2-S-(4-isothiocyanatobenzyl)-l-glutamate.

NOTA-NSC-GLU was synthesized by Nanchang Tanzhen Biotechnologies Co., Ltd, with >95% purity. ^18^F dissolved in water was passed through a preconditioned Sep-Pak QMA cartridge. Then, ^18^F was eluted from the QMA cartridge with 0.9% NaCl. Next, 90 μl of eluate was added to a vial containing 6 μl of 2 mM aluminum chloride, 5 μl of glacial acetic acid, 325 μl of acetonitrile, and 50 μg NOTA-NSC-GLU in 50 μl of deionized water. The resulting solution was incubated at 100°C for 10 min. The cooled crude reaction mixture was diluted with 10 ml of water and passed through a preconditioned C18 Sep-Pak cartridge. The radioactivity trapped in the C18 cartridge was eluted with 1.5 ml of ethanol. The ethanol solution was evaporated with an argon flow, and the final product was reconstituted in normal saline for further studies (Figure 1).

### Determination of Radiochemical Purity

The identity of [^18^F]AlF-NOTA-NSC-GLU was confirmed by analytical high performance liquid chromatography (HPLC) to determine its chemical purity. [^18^F]AlF-NOTA-NSC-GLU was injected into the HPLC to verify its components (Figure 1). Analytical HPLC was performed using an Agilent 1200 Series HPLC System equipped with a ZORBAX Eclipse XDB-C18 analytical column (4.66150 mm, 5 mm) using the flow rate of 1 ml/min. The gradient program started from 98% solvent A (0.1% TFA in water): 2% solvent B (0.1% TFA in MeCN) ramped to 90% solvent A: 10% solvent B at 8 min and ramped to 20% solvent A: 80% solvent B at 20 min. The elution profile was detected with an ultraviolet detector (Agilent Interface 35900E, Agilent Technologies, USA) at 210 nm and a B-FC-3200 high-energy PMT Detector (BioScan. Inc., Washington DC, USA).

### *In vivo* Biodistribution Studies

For *in vivo* biodistribution experiment, 20 HCC Hep3B tumor-bearing BALB/c nude mice were injected with 0.74 MBq (20 μCi) of [^18^F]AlF-NOTA-NSC-GLU in 100 μl of saline via the tail vein. Four animals were used at each time point. At 15, 30, 45, 60, and 90 min after injection, the distribution of the tracer in selected organs was evaluated. Organs of interest (the blood, brain, heart, lung, liver, spleen, kidneys, pancreas, stomach, intestine, muscle, and bone) were weighed and ^18^F radioactivity was counted with a γ-counter (GC-1200, USTC Chuangxin Co. Ltd. Zonkia Branch, China). All measurements were background-subtracted and decay-corrected to the time of injection, then averaged. The results were expressed as the percentage of injected dose per gram of tissue (%ID/g).

### Transport Assays

When HCC Hep3B cells were seeded into 24-well plates and reached the logarithmic proliferation phase, we performed the transport assays. The methods and transport mechanism of [^18^F]AlF-NOTA-NSC-GLU have been previously reported (Baek et al., 2013; Sun et al., 2017). In addition, each experiment was carried out in triplicate, averaged, and repeatedly conducted on three different days. Transport experiments were implemented in the presence and absence of Na^+^ (NaCl medium and choline chloride medium). For the competitive inhibition studies, we applied the following: α-(methylamino)isobutyric acid (MeAIB) for system A; serine (Ser) and L-glutamine (L-Gln) for system ASC; 2-amino-2-norbornane-carboxylic acid (BCH) for system L; L-glutamate (L-Glu) for system ${\text{X}}_{\text{C}}^{-}$ and ${\text{X}}_{\text{AG}}^{-}$; cystine (Cyss) for system ${\text{X}}_{\text{C}}^{-}$, and L-aspartic (L-Asp) and D-aspartic (D-Asp) for system ${\text{X}}_{\text{AG}}^{-}$. The concentration of the inhibitors was 15 mmol/l. Cells with [^18^F]AlF-NOTA-NSC-GLU (an average of 8 KBq per well) and the inhibitors were incubated at 37°C for 10 min. After washing three times with ice-cold NaCl or choline chloride medium, the activity of cells was measured by using a γ-counter. To further evaluate the role of system ${\text{X}}_{\text{C}}^{-}$ in the uptake of this agent, sulfasalazine, as an inhibitor of the ${\text{X}}_{\text{C}}^{-}$ system, was used in competitive inhibition experiments *in vitro*. The experiment was conducted as previously described [30] and divided into three concentrations: 100, 200, and 300 μM.

### *In vitro* and *in vivo* Stability and the Octanol–Water Partition Coefficient Study (logP)

For the stability tests *in vivo*, mice were injected with 11.1 MBq (300 μCi) of the [^18^F]AlF-NOTA-NSC-GLU (0.2 ml) via the tail vein. The mice were sacrificed at 1 h post-injection. Blood samples were collected from the eyeballs and then centrifuged (6,000 rpm for 4 min) to separate plasma and were used for the HPLC analysis.

Additionally, a sample of [^18^F]AlF-NOTA-NSC-GLU (1.48 MBq, 20 μl) dissolved in normal saline was incubated with 200 μl of fetal bovine serum at 37°C for 120 min. An aliquot of the serum sample was filtered through a 0.22-μm Millipore filter and was used for the HPLC analysis.

For the octanol–water partition coefficient study, 20 μl of [^18^F]AlF-NOTA-NSC-GLU (740 KBq, 20 μCi) in saline was added to an equal volume [octanol/phosphate buffered saline (PBS): 5/5 ml] mixture. The mixture was allowed to stand for complete phase separation prior to use by stirring in a vortex mixer for 2 min and centrifuging at 3,000 rpm for 5 min. Samples of 300 μl were taken from each layer and radioactivity was measured with a γ-counter. The logP value was calculated [logP = log10 (counts of octanol/counts of PBS)].

### Small-Animal PET/CT Imaging and Competitive Binding *in vivo*

Small-animal PET/CT imaging using an Inveon PET scanner (Siemens Healthineers, Erlangen, Germany) was performed following tail vein injection of 3.70–7.40 MBq (100–200 μCi) of [^18^F]AlF-NOTA-NSC-GLU in 100–200 μl of saline under pentobarbital sodium (50 mg/kg) anesthesia in tumor-bearing mice with HCC Hep3B cells (*n* = 3). The animals were kept fasting for at least 4 h before injection of the tracer and were visually monitored for breathing throughout the entire imaging period. Then, 10-min static PET image acquisition was performed at three time points (30, 60, and 90 min) post-injection. For a comparative study, the same mice (*n* = 3) were anesthetized with pentobarbital sodium (50 mg/kg) prior to scanning with [^18^F]FDG (4–6 MBq) at 60 min after IV injection. *In vivo* competitive binding experiments used respective models of L-Glu, L-Asp, and D-Asp [30% of lethal dose 50 (LD50), intraperitoneal injection, *n* = 3]. To perform the inhibition experiments for the ${\text{X}}_{\text{AG}}^{-}$ system, each inhibitor was injected 15 min prior to the injection of [^18^F]AlF-NOTA-NSC-GLU and small-animal PET/CT imaging was conducted at 30 min after administration of the tracer.

Imaging acquisition started with a low-dose CT scan (30 mAs), immediately followed by a PET scan. The CT scan was used for attenuation correction and organ localization. Image reconstruction was performed with the two-dimensional ordered subsets expectation maximization (2D-OSEM). The Inveon Research Workplace 4.1 software was used to draw regions of interest (ROIs) of 2 mm in diameter at the same section level of each PET/CT image. The radioactivity in each volume of interest was obtained from the mean pixel values and converted into megabecquerel per milliliter using a conversion factor. Assuming a tissue density of tissue was 1 g/ml, the ROIs were converted into megabecquerel per gram and then divided by the administered activity to obtain an imaging ROI-derived percentage of injected dose per gram of tissue. Finally, an imaging ROI-derived percentage of injected dose per gram of tissue and tumor-to-background relative uptake ratio were obtained.

### Incorporation of [^18^F]AlF-NOTA-NSC-GLU Into Protein

The method of determining the extent of protein incorporation of [^18^F]AlF-NOTA-NSC-GLU has been previously reported (Tang et al., 2017). Briefly, 400 μl (185–296 KBq) [^18^F]AlF-NOTA-NSC-GLU was added to the Hep3B cells and incubated at 37°C for 30 min. Upon removal of the radioactive medium, the cells were washed three times with ice-cold PBS (1.0 ml, pH = 7.4), separated by 0.5 ml of 0.25% trypsin, and resuspended in PBS. After centrifugation (13,000 rpm for 5 min), the supernatant was removed and the cells were suspended in 0.2 ml of Triton-X 100 (1%) prior to transferring into new vessels and adding 0.5 ml of 20% trichloroacetic acid (TCA). The mixture was kept in ice-cold water for 30 min and centrifuged at 13,000 rpm for 5 min. The supernatant was removed and the pellet was washed three times with ice-cold PBS. Radioactivity in both the supernatant and the pellet was counted with a γ-counter. Protein incorporation was calculated as the percentage of acid precipitable radioactivity. The experiment was repeated on three different days.

### Histochemical Studies

After the PET/CT scans, liver tissue and tumor samples were collected and histochemical studies were performed. Formalin-fixed, paraffin-embedded 3-μm-thick sections of tumor and liver were stained with hematoxylin and eosin (H&E). Immunohistochemistry (IHC) was performed using a previously reported method (He et al., 2000; Stoffel et al., 2004). Immunohistochemical staining of excitatory amino acid carrier 1 (EAAC1) was performed with a rabbit anti-EAACI monoclonal antibody (Abcam, 1:1,000).

### Statistical Analysis

Statistical analysis was performed using the Prism 6 Software (GraphPad Software, La Jolla, CA, USA). Data are presented as mean ± standard deviation (SD). Comparisons between conditions were made using unpaired, two-tailed Student's *t*-test. *P* < 0.05 was considered statistically significant, and *P* < 0.0001 was considered to indicate a meaningful difference.

## Results

### Radiosynthesis of [^18^F]AlF-NOTA-NSC-GLU

The overall radiochemical yield of [^18^F]AlF-NOTA-NSC-GLU from ^18^F was 29.3 ± 5.6% (*n* = 10) without decay correction within 20 min. The radiochemical purity of [^18^F]AlF-NOTA-NSC-GLU was >95%.

### Biodistribution Studies in BALB/c Nude Mice

**Figure 2:**
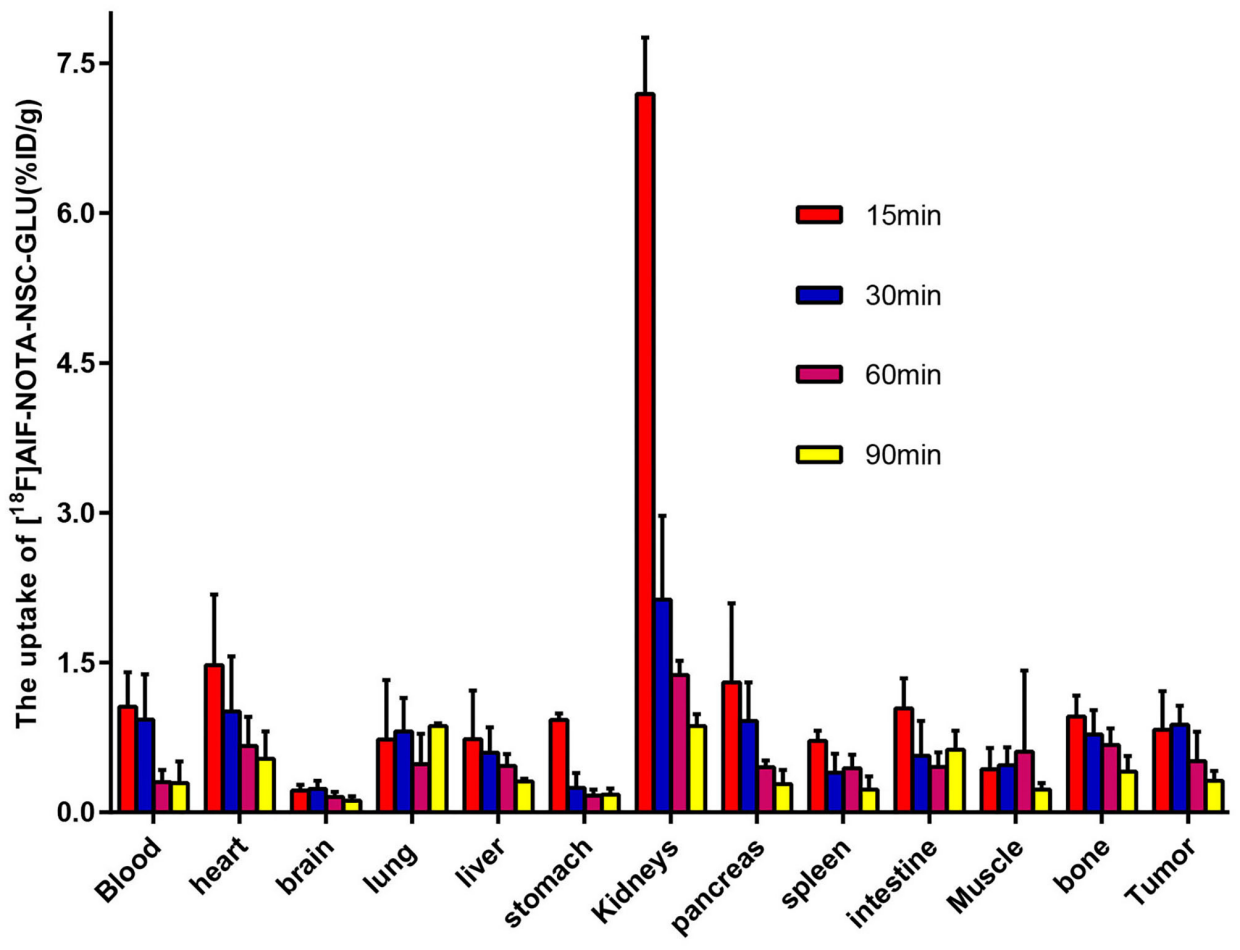
Biodistribution of [^18^F]AlF-NOTA-NSC-GLU in tumor-bearing mice.

The biodistribution of [^18^F]AlF-NOTA-NSC-GLU was evaluated in HCC Hep3B tumor-bearing BALB/c nude mice (Figure 2). The data revealed that the uptake of [^18^F]AlF-NOTA-NSC-GLU in the kidneys was high and decreased gradually from 7.19 ± 0.566% ID/g at 15 min to 0.86 ± 0.118% ID/g at 90 min post-injection. The stomach and intestine demonstrated a slightly high uptake of [^18^F]AlF-NOTA-NSC-GLU at 15 min, which decreased slowly at 30, 60, and 90 min. A moderate uptake of [^18^F]AlF-NOTA-NSC-GLU at 15 min post-injection was shown in the blood, heart, lung, pancreas, and bone with a relatively slow washout rate throughout the whole process. There were relatively low uptake levels of [^18^F]AlF-NOTA-NSC-GLU in other organs of interest (e.g., the liver, spleen, muscle, and brain), and the brain was the organ with the lowest uptake level (<1% ID/g).

### Competitive Inhibition Studies

**Figure 3:**
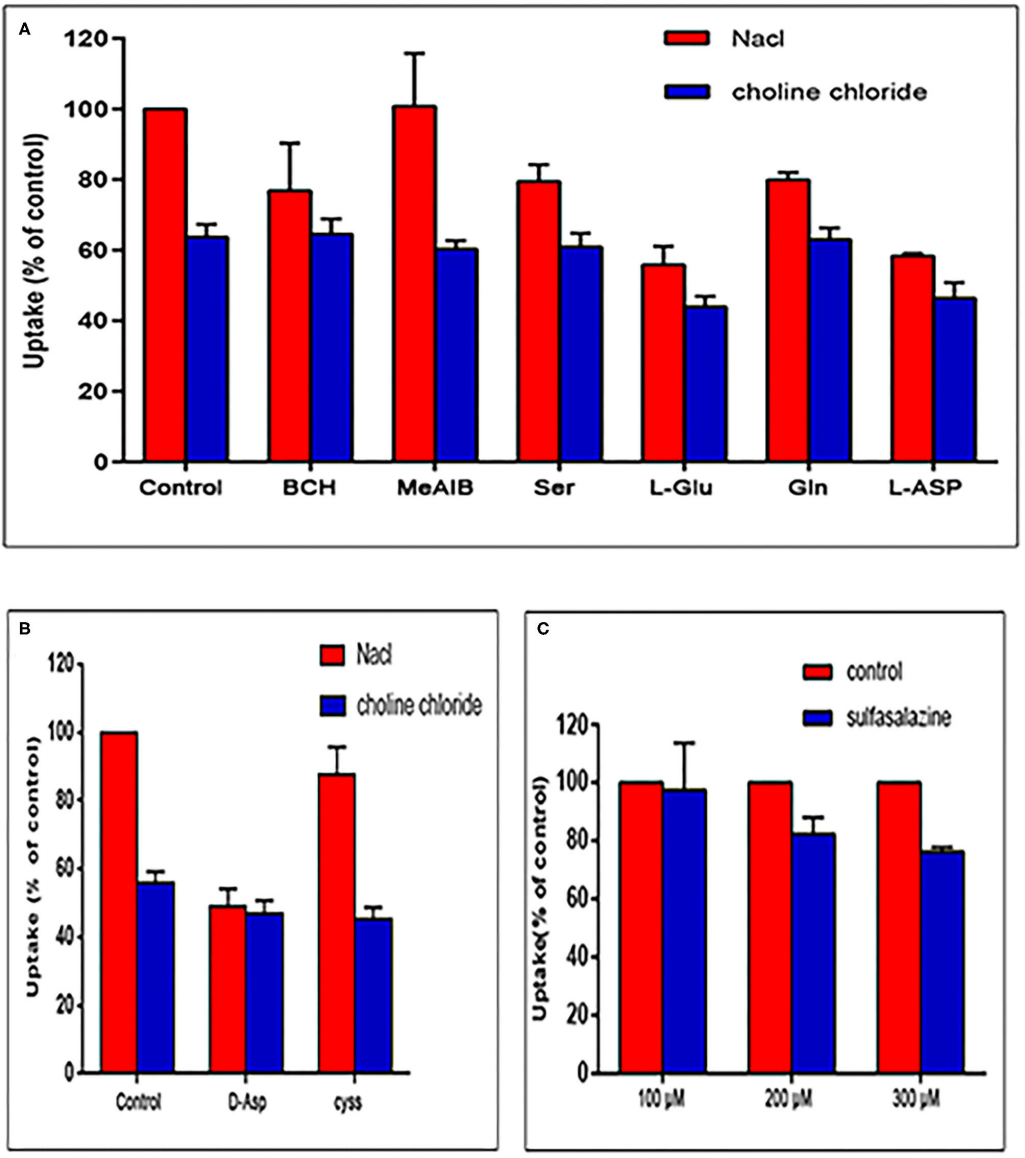
Uptake of [^18^F]AlF-NOTA-NSC-GLU in human HCC Hep3B cells in the competitive inhibition studies. **(A)** Uptake of [^18^F]AlF-NOTA-NSC-GLU in human HCC Hep3B cells in the presence or absence of Na^+^ by type of inhibitors. Values are given as percentage (mean ± SD, *n* = 15) of uptake in cells that were incubated with or without inhibitors, i.e., 2-amino-2-norbornane-carboxylic acid (BCH), α-(methylamino)isobutyric acid (MeAIB), serine (Ser), glutamine (Gln), L-glutamate (L-Glu), and L-aspirate (L-Asp) in NaCl (Na^+^-containing) or choline chloride (no Na^+^) buffer. **(B)** The inhibitors included D-aspirate (D-Asp) and cystine (cyss). **(C)** The inhibitor, sulfasalazine, was divided into three concentrations: 100, 200, and 300 μM.

The results of the competitive inhibition experiments are shown in Figure 3. In the presence of Na^+^, the uptake of [^18^F]AlF-NOTA-NSC-GLU was inhibited by 20.51 ± 4.77% and 20.07 ± 2.07% (*P* < 0.05) by substrate of system ASC, Ser, and Gln, respectively. The uptake of tracer was suppressed by BCH, an inhibitor of system B0^+^, by 23.2 ± 13.5% (*P* < 0.05). MeAIB, a specific inhibitor of system A, did not markedly suppress the uptake of [^18^F]AlF-NOTA-NSC-GLU. The addition of system ${\text{X}}_{\text{AG}}^{-}$ inhibitor L-Asp and L-Glu (an inhibitor for system ${\text{X}}_{\text{C}}^{-}$ or ${\text{X}}_{\text{AG}}^{-}$) inhibited the uptake of [^18^F]AlF-NOTA-NSC-GLU by 41.7 ± 0.76% and 44.14 ± 5.2%, respectively (*P* < 0.05). The specific inhibitor for system ${\text{X}}_{\text{AG}}^{-}$ and D-Asp suppressed the uptake of the tracer by 50.83 ± 5% (*P* < 0.05; Figure 3B). In addition, Cyss suppressed the uptake by 12.53 ± 8.23% (*P* < 0.05; Figure 3B).

In the absence of Na^+^, the addition of L-Glu and L-Asp decreased the uptake of [^18^F]AlF-NOTA-NSC-GLU by 30.95 ± 4.69% and 27.06 ± 6.88%, respectively (*P* < 0.05). However, other inhibitors (BCH, MeAlB, Ser, Gln, D-Asp, and Cyss) did not markedly inhibit the uptake of [^18^F]AlF-NOTA-NSC-GLU, which indicated that system ${\text{X}}_{\text{AG}}^{-}$ possibly played a dominant role in the transport of [^18^F]AlF-NOTA-NSC-GLU both in the presence and in the absence of Na^+^. As shown in Figure 3C, when HCC Hep3B cells were treated with the different concentrations of sulfasalazine, an inhibitor of system ${\text{X}}_{\text{C}}^{-}$, the uptake of this agent was inhibited by 17.6 ± 5.64% (200 μM) and 23.8 ± 1.5% (300 μM). Therefore, [^18^F]AlF-NOTA-NSC-GLU was primarily transported by the Na^+^-dependent system ${\text{X}}_{\text{AG}}^{-}$, with minor transportation by the Na^+^-dependent systems B0^+^, ${\text{X}}_{\text{C}}^{-}$, and ASC, with no involvement of the systems A transporter.

### Stability and the Octanol–Water Partition Coefficient Study (logP)

**Figure 4:**
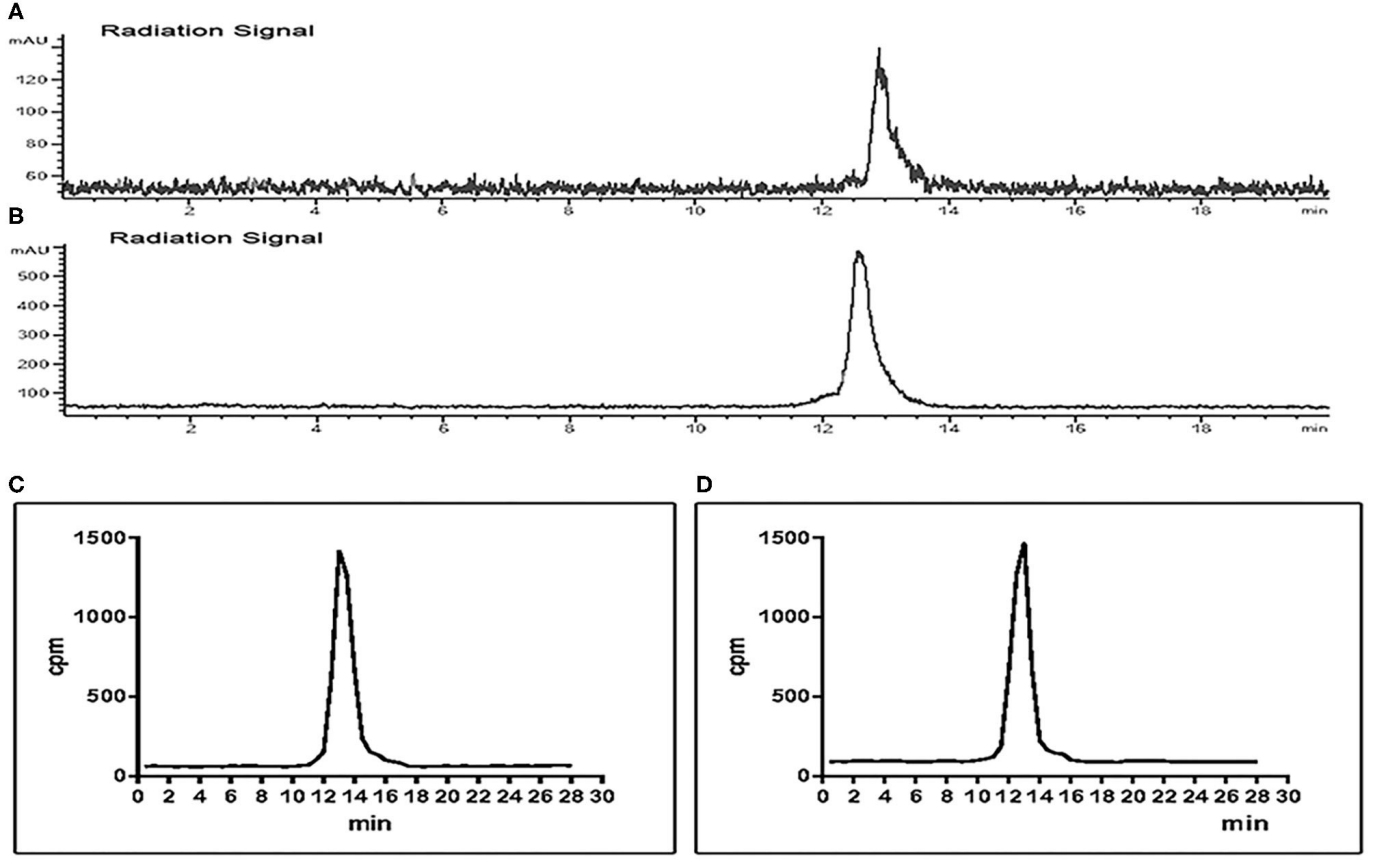
Chiral radio-HPLC of [^18^F]-AIF-NOTA-NSC-GLU (**C,D** test tube method*). (**A**) [^18^F]-AIF-NOTA-NSC-GLU-injected solution. (**B**) [^18^F]AlF-NOTA-NSC-GLU, co-cultured with fetal bovine serum for 2 h. (**C**) Post-injection of [^18^F]AlF-NOTA-NSC-GLU. (**D**) Plasma collected at 1 h post-injection of [^18^F]AlF-NOTA-NSC-GLU. *Test tube method was introduced as previously described (Liu et al., 2019). HPLC, high-performance liquid chromatography.

The stability test of [^18^F]AlF-NOTA-NSC-GLU was performed using radio-HPLC. Radio-HPLC analysis illustrated that over 95% of the [^18^F]AlF-NOTA-NSC-GLU remained intact after culturing with fetal bovine serum for 120 min at 37°C. The *in vivo* stability study in plasma showed that >95% of [^18^F]AlF-NOTA-NSC-GLU remained intact 1 h post-injection. Therefore, the stability of [^18^F]AlF-NOTA-NSC-GLU was relatively high both *in vivo* and *in vitro* (Figure 4). The lipophilic logP values of [^18^F]AlF-NOTA-NSC-GLU at pH 7.4 was −1.75 ± 0.05, which demonstrated that the tracer was hydrophilic.

### Small-Animal PET Imaging and Competitive Binding *in vivo*

**Figure 5:**
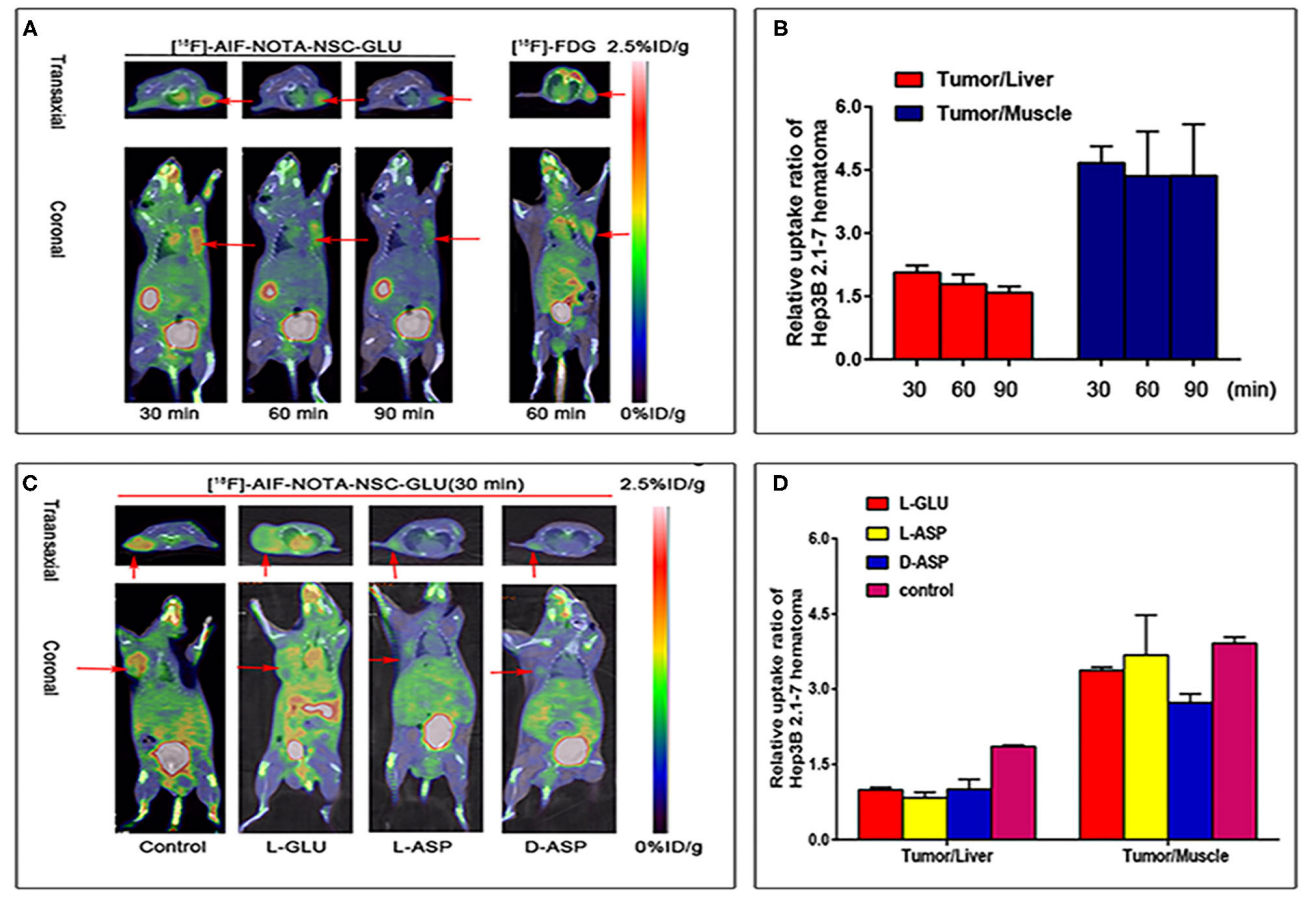
Small animal PET imaging of tumor-bearing (HCC Hep3B cell lines) nude mice. **(A)** PET/CT fusion images of tumor-bearing mouse static scans at 30, 60, and 90 min after the injection of [^18^F]AlF-NOTA-NSC-GLU, and images of the same tumor-bearing mouse after 60 min post-injection of [^18^F]FDG. **(B)** *T*/*L* and *T*/*M* ratios in tumor-bearing nude mice at different time points post-injection of [^18^F]AlF-NOTA-NSC-GLU. **(C)** PET/CT fusion images of tumor-bearing mouse static scans at 30 min after the injection of [^18^F]AlF-NOTA-NSC-GLU *in vivo* competitive binding. **(D)** The *T*/*L* and *T*/*M* ratios at 30 min post-injection of [^18^F]AlF-NOTA-NSC-GLU after the injection of L-glutamate (L-Glu), L-aspirate (L-Asp), and D-aspirate (D-Asp), respectively. PET/CT, positron emission tomography/computed tomography.

Small-animal PET/CT imaging was carried out with [^18^F]AlF-NOTA-NSC-GLU and with [^18^F]FDG in tumor-bearing (HCC Hep3B cell lines) nude mice (*n* = 3; Figure 5A). The data regarding uptake in organs of interest in the small animal PET imaging are shown in Table 1. High uptake of [^18^F]AlF-NOTA-NSC-GLU in the tumor was observed at 30 min post-injection. During the experiment, most radioactivity accumulations was found in the kidney and bladder, suggesting that the tracer was mainly cleared through the urinary system (Figure 5A). The uptake of [^18^F]AlF-NOTA-NSC-GLU in the tumor was 1.9 ± 0.057% ID/g, 1.33 ± 0.15% ID/g, and 0.99 ± 0.096% ID/g, respectively, at 30, 60, and 90 min post-injection; in the liver, however, it was 0.92 ± 0.025% ID/g, 0.75 ± 0.028% ID/g, and 0.62 ± 0.035% ID/g, respectively, at 30, 60, and 90 min post-injection (Figures 5B,C). The uptake of [^18^F]FDG in the tumor was 2.7 ± 0.12% ID/g at 60 min post-injection and in that the liver was 2 ± 0.015% ID/g. Hence, the tumor-to-liver uptake ratio for [^18^F]AlF-NOTA-NSC-GLU at 60 min post-injection was higher than that for [^18^F]FDG at 1 h post-injection (1.79 ± 0.228 vs. 1.37 ± 0.026, *n* = 3, *P* < 0.05). These results demonstrated the potential utility of [^18^F]AlF-NOTA-NSC-GLU as a PET tracer for HCC imaging. In addition, the tumor-to-liver and tumor-to-muscle uptake ratio for [^18^F]AlF-NOTA-NSC-GLU decreased after the injection of L-Glu, L-Asp, and D-Asp, respectively (Figure 5D; Table 2). The results of the competitive binding showed that the uptake of [^18^F]AlF-NOTA-NSC-GLU *in vivo* was also involved in the transport of the ${\text{X}}_{\text{AG}}^{-}$ system.

**Table 1:** The data of uptake in interest organs in small animal PET imaging (%ID/g).

	**30 min**	**60 min**	**90 min**
Tumor	1.90 ± 0.057	1.33 ± 0.150	0.99 ± 0.096
Liver	0.92 ± 0.025	0.75 ± 0.028	0.62 ± 0.035
Muscle	0.40 ± 0.030	0.31 ± 0.490	0.23 ± 0.050
Brain	0.16 ± 0.030	0.09 ± 0.010	0.15 ± 0.030
Heart	1.60 ± 0.030	1.10 ± 0.010	0.74 ± 0.030
Kidney	5.03 ± 0.730	3.43 ± 0.300	3.46 ± 0.450
Lung	0.86 ± 0.020	0.62 ± 0.010	0.48 ± 0.060
Bone	0.59 ± 0.080	0.49 ± 0.070	0.32 ± 0.060
Tumor/liver	2.06 ± 0.170	1.79 ± 0.228	1.58 ± 0.151
Tumor/muscle	4.67 ± 0.393	4.36 ± 1.060	4.36 ± 1.227

**Table 2:** The data of uptake in organs of interest in competitive binding *in vivo*.

	**Tumor (%ID/g)**	**Liver (%ID/g)**	**Muscle (%ID/g)**	***T*/*L***	***T*/*M***
[^18^F]AlF-NOTA-NSC-GLU	1.90 ± 0.057	0.92 ± 0.025	0.40 ± 0.030	2.06 ± 0.170	4.67 ± 0.393
[^18^F]AlF-NOTA-NSC-GLU after injection of L-Glu	4.90 ± 0.102	4.70 ± 0.103	1.86 ± 0.057	1.04 ± 0.040	2.62 ± 0.050
[^18^F]AlF-NOTA-NSC-GLU after injection of L-Asp	1.46 ± 0.152	1.76 ± 0.057	0.41 ± 0.061	0.83 ± 0.110	3.67 ± 0.802
[^18^F]AlF-NOTA-NSC-GLU after injection of D-Asp	0.19 ± 0.015	0.22 ± 0.005	0.07 ± 0.004	0.86 ± 0.072	2.73 ± 0.175

*L-Glu, L-glutamate; L-Asp, L-aspirate; D-Asp, D-aspirate; T/L, tumor-to-liver; T/M tumor-to-muscle*.

### Protein Incorporation

Protein-bound activity of [^18^F]AlF-NOTA-NSC-GLU in Hep3B cells indicated that ~1.25 ± 0.11% of the radioactivity was present in the acid precipitable fraction after co-incubating for 30 min. Hence, the uptake of [^18^F]AlF-NOTA-NSC-GLU in Hep3B cells is through AA transport rather than protein incorporation.

### H and E Staining and IHC

**Figure 6:**
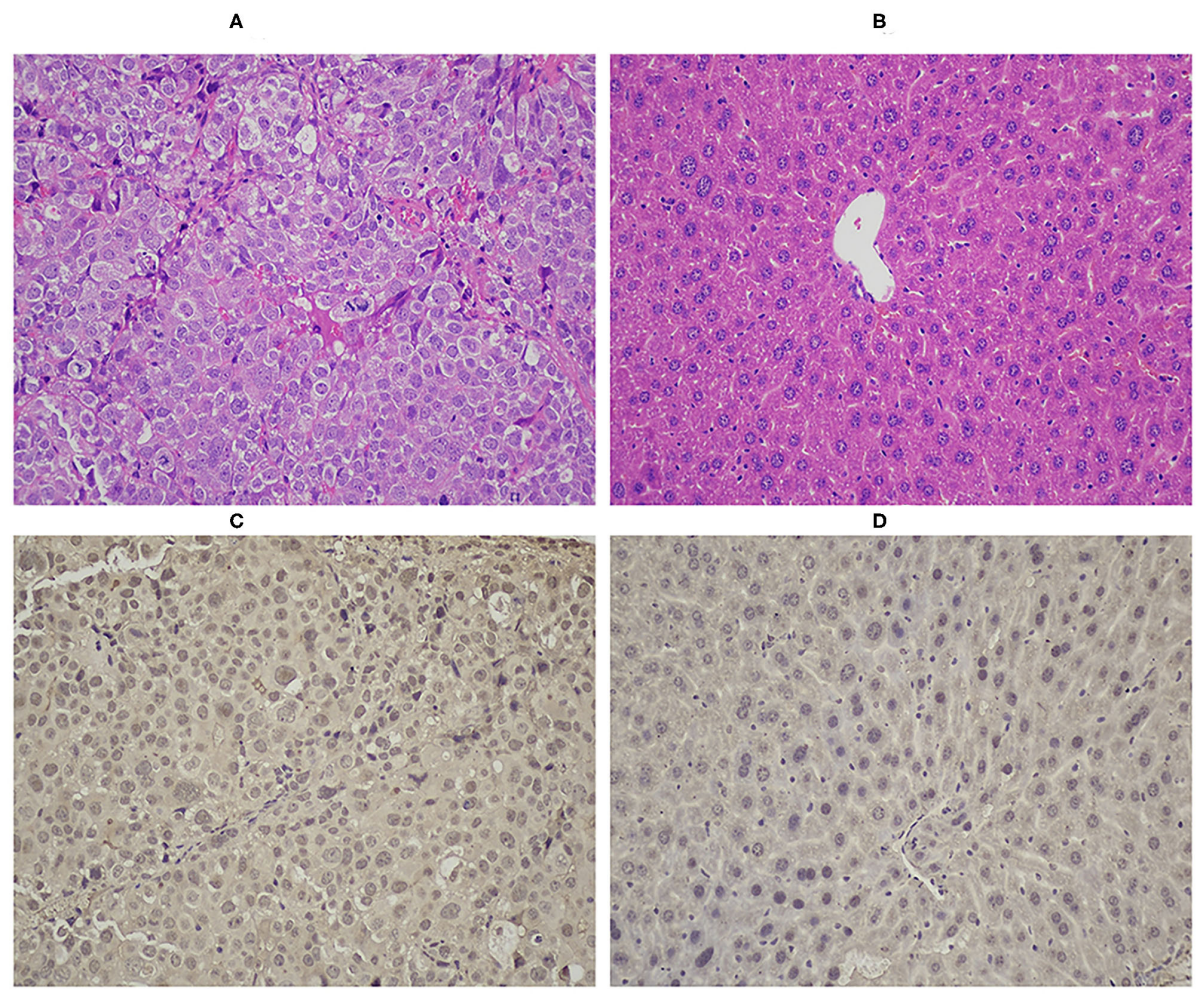
HandE staining of tumor (**A**, ×200) and of healthy liver (**B**, ×200). Immunohistochemical staining of EAAC1 in tumor (**C**, ×200) and in normal liver tissue (**D**, ×200). EAAC1, excitatory amino acid carrier 1; HandE, hematoxylin and eosin.

The results of the H&E staining are shown in Figures 6A,B. In Hep3B tumor, large cancer cells were observed by hematoxylin and eosin staining (Figure 6A). The results of immunohistochemical staining indicated that diffuse EAAC1 transporter staining was shown in Hep3B hepatoma (Figure 6C), while minimal EAAC1 staining was observed in normal hepatic tissue (Figure 6D), which suggested that the transport of [^18^F]AlF-NOTA-NSC-GLU in the Hep3B cell line was likely to involve glutamate transporter EAAC1.

## Discussion

[^11^C]Met is the most commonly used AA tracer in HCC imaging. Compared with [^18^F]AlF-NOTA-NSC-GLU, [^11^C]Met is susceptible to *in vivo* metabolism, complicating kinetic analysis (Ishiwata et al., 1989). PET imaging has been used in clinical applications (e.g., for detection, diagnosis, and identification of distant metastasis and effect monitoring) for decades. Although glucose metabolism plays an important role in tumor cell growth, glutamine metabolism is considered second only to glucose in importance in tumor (Li and Zhang, 2016). [^18^F]FPGLU (Liu et al., 2017; Sun et al., 2017), (2S,4R)-4-fluoro-l-glutamate ([^18^F](2S,4R)4F-GLU) (Ploessl et al., 2012), and (4S)-4-(3-[^18^F]fluoropropyl)-l-glutamate ([^18^F]FSPG) (Chopra, 2004), as ^18^F-labeled glutamate and metabolic imaging agents, have been used in tumor imaging studies. [^18^F]FPGLU and [^18^F]FSPG are synthesized via a two-step process. In addition, [^18^F](2S,4R)4F-GLU is synthesized through a multi-step process. Hence, the time-consuming and multi-step synthetic methods prompted the search for novel ^18^F-labeled glutamate imaging agents. The method of using the ^18^F-fluoride-aluminum-NOTA complex for labeling peptides, which could simplify the labeling procedure and reduce the time for radiosynthesis, was reported by McBride et al. (2010) (Laverman et al., 2010). Here, we present the successful synthesis of a new ^18^F-labeled glutamate imaging agent via the ^18^F-fluoride-aluminum-NOTA complex for labeling peptides. [^18^F]AlF-NOTA-NSC-GLU, as a novel glutamate imaging agent, was prepared in 25 min via a one-step synthetic method. Comparisons of [^18^F]AlF-NOTA-NSC-GLU, [^18^F]FPGLU, [^18^F]FSPG, and [^18^F](2S,4R)4F-GLU are shown in Table 3.

**Table 3:** The comparisons of [^18^F]AlF-NOTA-NSC-GLU, [^18^F]FPGLU^*^, [^18^F]FSPG^*^, and [^18^F](2S,4R)4F-GLU^*^.

	**[^**18**^F]AlF-NOTA-NSC-GLU**	[^**18**^F]FPGLU^*^	[^**18**^F]FSPG^*^	[^**18**^F](2S,4R)4F-GLU^*^
Radiochemical yield	29.3 ± 5.6%	20 ± 3%	40–63%	8.4 ± 3.4%
Radiochemical purity	>95%	98%	>92%	>95%
Specific activity (GBq/μmol)	25 ± 5	60 ± 8	>18.2	–
Total synthetic time (min)	20	35	41–51	30
Process	One-step	Two-step	Two-step	Multi-step
Transport mechanism	Na^+^-dependent system ${\text{X}}_{\text{AG}}^{-}$	Na^+^-dependent system ${\text{X}}_{\text{AG}}^{-}$ and ${\text{X}}_{\text{C}}^{-}$	System ${\text{X}}_{\text{C}}^{-}$	Na^+^-dependent system ${\text{X}}_{\text{AG}}^{-}$ and ${\text{X}}_{\text{C}}^{-}$
Bone uptake (ID/g)	<1%	<1%	<1%	>1%

* *Data were first published by Chopra (2004), Lieberman et al. (2011), Ploessl et al. (2012), and Liu et al. (2017)*.

A biodistribution study of this tracer demonstrated that the kidney had the highest accumulation of all organs at 15 min after injection, suggesting that the renal–bladder system was the main excretory route. Although the uptake in the stomach and intestine was slightly high at 15 min post-injection, other tissues showed relatively low uptake during the entire observation period, suggesting that the tracer had a low background signal *in vivo*. There were relatively low uptake levels of this agent in the bone (**<**1% ID/g) during the entire observation period, suggesting no defluorination of [^18^F]AlF-NOTA-NSC-GLU *in vivo*. The biodistribution results were also confirmed by small-animal PET imaging. Surprisingly, the lowest levels of activity were observed in the brain, like other ^18^F-labeled glutamate imaging agents. On the one hand, this suggests that [^18^F]AlF-NOTA-NSC-GLU could be a potential agent in brain tumor imaging: further studies are needed to determine its suitability for this. On the other hand, it also indicates that [^18^F]AlF-NOTA-NSC-GLU will not be able to access the brain via the blood–brain barrier.

The results of *in vitro* experiments also demonstrated satisfactory stability and hydrophilicity of [^18^F]AlF-NOTA-NSC-GLU. Moreover, 95% of [^18^F]AlF-NOTA-NSC-GLU was preserved intact 1 h post-injection *in vivo*, which demonstrated that the product was also relatively stable *in vivo*.

Like many ^18^F-labeled glutamate imaging agents, [^18^F]AlF-NOTA-NSC-GLU is almost not incorporated into the protein, indicating that it also can reflect the AA transport rate in tumors. Furthermore, AAs generally enter cells via membrane-associated carrier proteins, and malignant tumor cells accumulate AAs owing to the increased expression of AA transporters (Ishiwata et al., 1993). To investigate the transport mechanism involved in the uptake of [^18^F]AlF-NOTA-NSC-GLU, we conducted a series of competitive inhibition studies in Hep3B cells using specific inhibitors for system A, ASC, L, ${\text{X}}_{\text{C}}^{-}$, and ${\text{X}}_{\text{AG}}^{-}$ (Kong and Yang, 2012). The competitive inhibition results suggested that transport of [^18^F]AlF-NOTA-NSC-GLU was mainly mediated through the Na^+^-dependent system ${\text{X}}_{\text{AG}}^{-}$. In addition, minor system ${\text{X}}_{\text{C}}^{-}$ and Na^+^-dependent systems B0^+^ and ASC were partly involved in the transport of [^18^F]AlF-NOTA-NSC-GLU, with almost no involvement of systems A. [^18^F]FSPG has been used previously for HCC imaging via the system ${\text{X}}_{\text{C}}^{-}$ system (Baek et al., 2013). In addition, the Na^+^-dependent system ${\text{X}}_{\text{AG}}^{-}$ and Na^+^-independent system ${\text{X}}_{\text{C}}^{-}$ were involved in the transport of [^18^F](2S,4R)4F-GLU and [^18^F]FPGLU (Ploessl et al., 2012), but system ${\text{X}}_{\text{C}}^{-}$ plays a more primary role in the transport of [^18^F](2S,4R)4F-GLU (Ploessl et al., 2012). System ${\text{X}}_{\text{C}}^{-}$, as a cystine/glutamate antiporter, can use extracellular cystine to exchange for intracellular glutamate (Bannai and Ishii, 1988). In addition, SLC1A5 (ASCT2), SLC7A5 (LAT1), SLC7A11 (xCT), and SLC6A14 (ATB^0+^) have been shown to be positively expressed in cancer. It has been found that xCT, a member of the system ${\text{X}}_{\text{C}}^{-}$ transporter, is actively expressed in HCC patients (Wada et al., 2018). Cyss, a substrate of the ${\text{X}}_{\text{C}}^{-}$ system, did not markedly inhibit the uptake of this agent. Hence, sulfasalazine, a specific inhibitor of XCT-mediated cystine transport, was used in this experiment (Patel et al., 2019). Thus, system ${\text{X}}_{\text{C}}^{-}$ also involved and played a minor role in the [^18^F]AlF-NOTA-NSC-GLU uptake. The transport mechanisms of [^18^F]AlF-NOTA-NSC-GLU were not fully consistent with other ^18^F-labeled glutamate imaging agents, possibly due to the modification of the NH_2_ group in glutamate.

Excitatory amino acid transporters (EAATs), including EAAT1, EAAT2, EAAT3 (EAAC1), EAAT4, and EAAT5, are plasma membrane glutamate transporters in humans (Olivares-Banuelos et al., 2019). In addition, recent research has demonstrated that EAAC1, an important member of system ${\text{X}}_{\text{AG}}^{-}$, is highly expressed in several human glioma cell models (Palos et al., 1996) and human PC-3 prostate tumor cells (Hermanson and Blomqvist, 1997). We also found high expression of EAAC1in HCC Hep3B-bearing mice with IHC staining, while minimal EAAC1 staining was observed in normal hepatic tissue. Thus, we tentatively conclude that the higher uptake of [^18^F]AlF-NOTA-NSC-GLU relative to [^18^F]FDG in HCC may be a result from the high expression of the ${\text{X}}_{\text{AG}}^{-}$ transporter.

PET images of the HCC tumor-bearing models showed that the tumor uptake of radioactivity occurred at 30 min after injection. The tumors were clearly visible, with good contrast to the liver. Moreover, the *T*/*L* ratio for [^18^F]AlF-NOTA-NSC-GLU was higher than that for [^18^F]FDG at 60 min post-injection. Possible explanations include the relatively high accumulation of [^18^F]AlF-NOTA-NSC-GLU in tumors and the low accumulation in healthy liver tissues. The system ${\text{X}}_{\text{AG}}^{-}$ inhibitors L-Glu, L-Asp, and D-Asp were used for *in vivo* competitive binding. Surprisingly, the tumor-to-liver and tumor-to-muscle uptake ratio for [^18^F]AlF-NOTA-NSC-GLU was reduced after blocking the ${\text{X}}_{\text{AG}}^{-}$ system, which was consistent with the competitive inhibition studies *in vitro*. Thus, the results indicated that system ${\text{X}}_{\text{AG}}^{-}$ was the main transporter for [^18^F]AlF-NOTA-NSC-GLU uptake not only *in vitro* but also *in vivo*. Taken together, the results were very encouraging for this new PET tracer, but further investigations are needed to confirm the suitability of its clinical application. At the same time, we can see a very fast accumulation of the ligand in the targeted tissue, but the *T*/*L* or *T*/*M* ratio decreased during the experiments. The real specific interaction to achieve a stable accumulation is missing between the tracer and the targeted tissue (Pellegrini et al., 2013). Hence, an orthotopic HCC model and more HCC models (SMCC-7721 cells and HepG2 cells) should be used to further evaluate this agent.

## Conclusion

We found that [^18^F]AlF-NOTA-NSC-GLU, with good radiochemical yield and purity, is an AA imaging agent that can be synthesized conveniently. Preliminary results demonstrated that [^18^F]AlF-NOTA-NSC-GLU was superior to other ^18^F-labeled glutamate agents, owing to the one-step method. Furthermore, it also showed a good target-to-background ratio in HCC imaging. The Na^+^-dependent system ${\text{X}}_{\text{AG}}^{-}$ played a more dominant role in the uptake of [^18^F]AlF-NOTA-NSC-GLU *in vitro* and *in vivo* and showed good stability both *in vitro* and *in vivo*. Therefore, [^18^F]AlF-NOTA-NSC-GLU can potentially be used as an AA PET probe for HCC imaging. Further biological evaluation of [^18^F]AlF-NOTA-NSC-GLU is needed to confirm its applicability.

## Data Availability Statement

The original contributions presented in the study are included in the article/supplementary material, further inquiries can be directed to the corresponding authors.

## Ethics Statement

The animal study was reviewed and approved by The Institutional Animal Care and Utilization Committee (IACUU) of the First Affiliated Hospital, Sun Yat-Sen University (approval number 2018033).

## Author Contributions

LL and XX performed the data analyses and wrote the manuscript. SS and SL contributed significantly to analysis and manuscript preparation. YX, HM, and GY helped perform the analysis with constructive discussions. GT and DN contributed to the conception of the study. All authors contributed to the article and approved the submitted version.

## Conflict of Interest

The authors declare that the research was conducted in the absence of any commercial or financial relationships that could be construed as a potential conflict of interest.
